# Older People’s Long-Term Care Preferences in China: The Impact of Living with Grandchildren on Older People’s Willingness and Family Decisions

**DOI:** 10.3390/ijerph191912455

**Published:** 2022-09-29

**Authors:** Tongbo Deng, Yafan Fan, Mengdi Wu, Min Li

**Affiliations:** 1 School of Public Administration, South China University of Technology, Guangzhou 510641, China; 2 College of Economy and Trade, Zhongkai University of Agriculture and Engineering, Guangzhou 510220, China; 3 School of Business Administration, South China University of Technology, Guangzhou 510641, China

**Keywords:** older people, institutional care preference, family members’ attitudes, gender, social exchange theory, the demonstration effect

## Abstract

The purpose of this paper was to better understand the long-term care preferences of older people based on intergenerational demonstration effects and social exchange theory, derived from the literature on intergenerational family relationships. The authors relied on the 2014 China Longitudinal Ageing Social Survey database to test the study hypotheses. The results indicated that living with grandchildren was negatively related to the institutional care preferences of older people. Family members’ attitudes and older people’s life satisfaction significantly mediated the relationship between living with grandchildren and their institutional care preferences. Gender and marital status had potentially diverse effects on institutional care preferences. Therefore, in the context of China’s culture of filial piety, social exchange, and intergenerational demonstration, motivation may help foster intergenerational exchange and reciprocity in eldercare arrangements.

## 1. Introduction

China has become one of the fastest aging countries globally with an increased demand for long-term care (LTC). This tendency is anticipated to lead to increasing pressure on the state to offer options for families, and on markets for aged long-term care for older people [1]. The Chinese government has issued policy documents supporting LTC, but it is unclear whether these policies align with older people’s care preferences. The aging of the population brings with it increased life expectancy and a high frequency of chronic illness. The low healthy life expectancy associated with chronic illness has resulted in a high demand for LTC [2,3]. As of 2021, older people made up 14.2 percent of the total population (2.06 million people) in China. According to an investigation in 2018, China’s average life expectancy was 77 years, while the average healthy life expectancy was just 68.7 years, which is a 10-year difference [2]. The demand for LTC is increasing as the population ages, and as chronic and degenerative illnesses become more prevalent. Furthermore, China has a high disability rate. The total number of people with disabilities in China has increased from 51.64 million in 1987 to 85 million in 2020, with 44.16 million people over 60 years of age nationwide, accounting for 53.24% of the total number of people with disabilities nationwide in China [4,5]. The formal care system in China is still underdeveloped. Recognizing these challenges, the Chinese central government has formulated a series of policies to increase the capacity of formal care [6]. The Chinese government has implemented a series of policies to support the development of institution-based LTC as one of the key guidelines put forth by the 13th Five-Year Plan (2016–2020). The State Council published directives on promoting this fledgling industry, the development plan on home care services for older people, as part of a concerted effort to meet the growing demand for long-term care [2]. However, few studies have focused on the attitudes of older people and their family toward eldercare patterns.

Although older people’s opinions have been highlighted as a critical determinant in attaining a higher engagement of older people in their own care, few studies have explored the ideal dimension of residential and LTC preferences [3]. Generally, there are two types of LTC, which are referred to as informal and formal care. Family members or close relatives provide informal care that requires low-level skills to frail, older family members, such as children who perform personal maintenance for their parents at home, while institutional or social systems such as nursing homes supply formal care [7]. Therefore, examining the preferences of older people regarding their care is of paramount significance in terms of improving the LTC system. Older people in China prefer to live at home, and adult children have historically been the dominant long-term caregivers in China [8]. While many older Asian people would rather be cared for by family members, several studies have found that today’s older Asian people value self-reliance and privacy more than ever before and are less willing to burden their family members [6]. Aside from these shifting views, today’s seniors have more financial resources and are generally healthier and more educated than their ancestors, all of which influence the type of care they require or choose [9]. Moreover, changes in demographics and family structure have significantly weakened the function of family care and the form of intergenerational transfer in several ways [2]. On the one hand, the implementation of the one-child policy in the 1990s, the two-child policy in 2016 and the three-child policy in 2021, led to a situation that is commonly referred to as “422/423” families, meaning that there are four grandparents, two parents, and two or three children in a family. On the other hand, the mobility of adult children may also create a lack of caretakers [1,2]. All of these factors have challenged the tradition of eldercare being provided by adult children. According to the substitution model, formal care typically happens when significant elements of informal care networks are lacking or when there are increasing care demands that the family cannot manage on its own [10]. However, it is unclear whether an older person’s family situation and living arrangements have an impact on their institutional care preferences [11].

Accordingly, our first aim was to determine the institutional care preferences of older people. In recent decades, choices for informal care have led to a low demand for formal care in China, especially for people with functional limitations [12]. On the one hand, evidence has suggested that the family home is not always the best place for eldercare because of scarce supportive resources, the lack of suitable informal caregivers, and the possibility of financial, verbal, and even physical abuse [13]. On the other hand, recent reports have revealed that publicly and privately owned nursing homes have rapidly emerged in major urban centers in China [2]. The institutional pension rate of the Chinese aging population is rising rapidly, possibly due to increased demand [8]. However, the diminishing usage of institutional care is perplexing, given the aging of the population and the resulting increased demand for care [9]. In terms of the development of institutional care and the challenges of family care, the LTC preferences of older people are essential in assessing the future development of LTC.

The second aim of this study was to examine the reasons for family members’ opinions on long-term eldercare in terms of reciprocal exchanges of support and demonstration effects. A previous study mainly focused on the effect of two important aspects, economic burdens, and health conditions, on the choice of informal or formal LTC. In regard to formal LTC expenditures, Medicare offers little or no LTC insurance coverage in China, and people must pay out of pocket for formal care, which may be a major obstacle for many older people. Indeed, informal care is regarded to be vastly cheaper than formal care. The current literature states that along with the shift in views, older people today also tend to be wealthier or attain additional financial support from their adult children, which may expand the family budget, affecting the optimal choice of LTC arrangements [14]. In consideration of health conditions, the impact of family economic status on living arrangements may vary depending on the older parents’ health or expected health circumstances [14,15]. This paper did not repeat previous studies that focus on the health and economic factors affecting LTC preferences, and instead considered that China is experiencing a period of transformation in terms of family structure and social norms. It is essential to identify the motives for decisions made within the family regarding eldercare [16]. Intergenerational co-residence is still common in China, although there has been a recent downward trend, while institutional care living is still rare for obvious reasons. The issue that has been overlooked is whether long-term eldercare preferences within a family are motivated by exchange or intergenerational demonstration. To this end, we concentrated on three generations, namely, grandparents (G), parents (P), and their children (C). We attempted to determine the effects of demonstration and social exchange on the eldercare preferences of individuals in these three generations, and whether adult children want to manipulate older people’s care preferences through social exchange and demonstration effects.

The third aim of this study was to investigate whether the institutional care preferences of older parents are valid to the underpinnings of the intergenerational exchange effect through gender or marital status difference effects. Studies have shown that spouses always play an important role in the provision of informal care [17]. Naturally, an older person without a spouse lacks the opportunity to be cared for. The difference in care preferences between single individuals and older adults with spouses is important. Moreover, this paper also examined the gender dimension of intergenerational exchanges of institutional care preferences for older adults. The focus was on the reciprocity exchange and differences in responsibilities among the three generations and their connections in different genders or marital statuses due to the cultural significance of Confucian tradition. It is essential to analyze the effect of traditional gender roles and filial piety when discussing intergenerational reciprocity in China [18].

Our study made three primary contributions. First, we defined living with grandchildren and family members’ attitudes as key factors in determining single older people’s preferences regarding institutional care. To date, little research has examined the care preferences of older people based on intergenerational demonstrations. Expanding the understanding of eldercare arrangements and family members’ attitudes is critical for forecasting and responding to the demands for formal institutional eldercare [19]. Second, we contributed to the growing literature on the effects of living with grandchildren on family members’ attitudes toward their older family member’s institutional care preferences, by introducing social exchange and intergenerational demonstration motivation as a relevant contingency factor for an older person’s selection of institutional care. Third, we proposed a new hypothesis that links intergenerational living arrangements, gender and marital status differences, in intergenerational caregiving attitudes, and institutional care preferences.

## 2. Model and Hypothesis Development

### 2.1. Social Exchange Theory and Demonstration Effect

In later life, care and residential preferences are inevitably related to both the family situation and cognitive needs. Our paper examined attitudes toward older people’s care preferences within three familial generations and across two strategic motives, the social exchange motive of the grandparent (G) generation and the demonstration motive of the parent (P) generation [20].

The intergenerational relationship theory of the family system stresses the importance of interdependence among family members [21]. Grandparents, parents, and children, all play a crucial role in deciding the preference for institutional care in intergenerational cognitive support. If intergenerational exchange guides care across generations, then aging itself can be regarded as an exchange of interdependence [21]. The relationships among these three generations were embedded within this research system to investigate the effect of a complete kinship network on formal institutional care preferences.

Social exchange theory is an influential approach to studying intergenerational family obligations [22]. Social exchange theory regards social life as a sequence of transactions to promote social connections with people [23]. Resources are exchanged through the process of reciprocity. Intergenerational social exchange has been established as a helpful paradigm that guides family members in their exchange relationships [16]. Therefore, intergenerational social exchanges are treated as an investment strategy. Parents offer support to their children when they are younger and expect to gain resources back from their children’s families in the future [24]. Different generations in a family are willing to exchange various goods and services directly and simultaneously [21]. However, older family members might find it difficult to reciprocate when they receive support, and intergenerational social exchanges depend on the motivations throughout different stages of life, as opposed to time-limited exchanges [25]. Family intergenerational exchanges maintain bidirectional, dynamic, and continuous transmissions [24]. As resources and power flow between generations, current support helps motivate future intergenerational exchanges [18].

The demonstration effect in intergenerational transfers aims to explain the care, companionship, and other types of support and care, that adult children offer to their parents [20,26]. To do this, the analysis of intergenerational interaction is expanded from two to three generations, with a focus on the possibility that a child’s behavior is influenced by parental example, and that parents take advantage of their children’s learning potential by showing care and attention to their own parents, while the latter are present to watch and are impressionable [27]. According to the demonstration effect, adult children, who are the middle generation, are motivated by self-interest [28]. More concretely, this paper considered a family to consist of three overlapping generations with two dependent generations: grandparents (G); parents (P); and minor children (C) [29]. Forward-looking parents (P) provide obvious help and support to their parents, the grandparents (G), in the presence of their minor children (C) to set an example for them. Through this example, parents (P) hope to encourage their minor children (C) to replicate their behavior by caring for and helping them in later life, according to their demonstration [26,27,28,29]. According to the demonstration effect, adult parents (P) create a norm of manipulating their children (C) to perform the same for them (P) as they age. This is also referred to as preference shaping. Some research has provided additional evidence supporting the existence of the family demonstration effect [26,27].

### 2.2. The Main Effect of Living with Grandchildren on Institutional Care Preferences

According to social norms [21], the middle generation (P) wants to co-reside with a single older person (G) for companionship and convenience of care [26]. According to the altruistic model, family members are in a web of mutual support to meet the family’s collective needs [24]. Driven by altruism and self-sacrifice, the single older person emphasizes obligations and responsibility to the offspring. Thus, single older individuals are willing to live with their minor grandchildren and offer caregiving to decrease the pressure on parents when they cannot fulfil these obligations due to work or other challenges [21]. The evidence has also shown that multigenerational households are significantly and positively related to grandparents providing care for their minor grandchildren [28].

Social exchange theory can also explain other important motivations, in addition to altruism, for a single older person living with their grandchildren. Intergenerational reciprocation may last for many years and does not necessarily exist in the form of immediate exchanges [21]. Monserud (2008) demonstrated that earlier care and interactions between grandparents and grandchildren result in more robust, supportive relationships later in life [30]. Childcare provided by grandparents is an investment in intergenerational resource exchange. The goal is to access support and attention from family members in later life [21].

**Hypothesis** **1** **(H1).** *Living with grandchildren is negatively related to older people’s institutional care preferences.*

### 2.3. The Mediating Effect of Family Members’ Attitudes

We first postulated that living with grandchildren fosters a demonstration effect, reducing parents’ willingness to send grandparents to a nursing home. Living with grandchildren positively affects intergenerational support from parents to grandparents because parents want to set an appropriate example of family caregiving for their children [31,32]. Generally, parents with minor children may provide more care for grandparents than those without small children because the grandchildren in these cases have an opportunity to learn from their parents’ examples of caregiving [29]. Previous research has also confirmed this conclusion, as those who experienced co-residing with a grandparent in their early lives tended to have a favorable opinion of co-residing with their own older parents [31]. A similar consideration applies when deciding to place a grandparent in a nursing home, where parents expect their children to imitate them [16,27].

Our study predicted that the attitudes of older people’s adult children significantly determined their preferences for living and care arrangements. In general, the head of the family, the middle generation of the three generations, has higher bargaining power and decision-making power surrounding eldercare. Prior research has argued that adult children, adult grandchildren, and other close family members, are the most active participants in selecting eldercare arrangements, severely impeding the ability of older people to choose for themselves [14]. The middle generation, the parents (P), also play a vital role in the intergenerational exchange; their influence supports the relationships between the grandparents and grandchildren [30]. The parental generation holds a norm-setter role, a mediating position in promoting the relationships between grandparents and grandchildren [33]. Parents (P) who have minor children (C) would be more eager to live with their single older parents (G), who can help with child-rearing, as per the exchange incentive [29].

According to social exchange theory, intergenerational trusting relationships transfer between older parents and their adult children. Parents provide support and make decisions on behalf of their children in their youth, transmitting attitudes and will from parent to child [34]. According to reciprocity, the intergenerational transmission of familial trust leads older parents to expect an upward flow of support from their children [14]. Older parents are willing to rely on and trust their adult children in their later years, resulting in the implicit compliance of older people with their children’s care choices [14]. Hence, we formulated the following hypothesis:

**Hypothesis** **2** **(H2).** *Family members’ attitudes toward sending older people to nursing homes mediate the relationship between living with grandchildren and older people’s institutional care preferences*.

### 2.4. The Mediating Effect of Life Satisfaction

Grandparents providing care for their grandchildren have an impact on the grandparents’ psychological well-being, including their life satisfaction [21]. The life satisfaction of grandparents may be derived from their duties as grandparents, but the results of current studies on grandparents providing care and their life satisfaction have been inconsistent. Some studies discovered that grandparents perceive caring for their grandchildren as a burden that reduces their life satisfaction [35], while others considered the experience gratifying or conditional based on certain conditions, as discussed later.

Reciprocal exchange motivation, which is based on cost/benefit economic principles, is frequently used to explain the relationship between intergenerational support and adult life satisfaction in later life, implying that people will have higher levels of well-being when the support they receive exceeds the support they provide [35]. Some studies have found that grandparents perceive great roles in their relationships with their grandchildren and hence build positive identities [36]. Grandchild caregiving may boost grandparents’ moods due to the intrinsic reward of assisting a loved one and contributing to sentiments of family continuity and well-being, which assists older people in finding meaning and fulfilment and is significantly linked to their life satisfaction [21]. In addition, grandparents often look after their grandchildren in Chinese traditional culture, and the grandparenting role reflects social and cultural norms and expectations based on role theory [21,37]. Thus, grandparents who adapt their role in the family and the growth phases of grandchildren associate intergenerational relationships with caring and love, helping establish a positive identity and leading to psychological satisfaction [37].

Based on the above analysis, grandparents in China are an important part of a multigenerational structure in which resources are shared, and grandparent caregiving is generally conducted as part of a broader coordinated strategy within a family [21]. Specifically, life satisfaction is a conscious cognitive appraisal of one’s life, based mostly on the available longitudinal information about one’s life and an individual’s own criteria for what characterizes a good life [38]. Grandparents in China who participate in the culturally significant activity of caring for grandchildren may find considerable joy in this vital family duty [21]. The life satisfaction of grandparents thereby improves the preference for family care, reducing institutional care preferences. Based on the above reasoning, we hypothesized the following:

**Hypothesis** **3** **(H3).** *Life satisfaction mediates the relationship between living with grandchildren and older people’s institutional care preferences*.

### 2.5. The Heterogeneous Effect of Older People’s Marital Status

We also examined the potential heterogeneous effects of marital status. Prior research has shown that the family structure, specifically the relationships with children and grandchildren, has a prominent significance in preferences for future care management [39]. In terms of marital status, older people without spouses tend to be lonely and desire the company of their families [40], which is primarily because their spouses and children are generally their primary caregivers. Moreover, single older people who reside with their children are less likely to receive proper institutional care, as they are likely to have a more extensive informal home care network within their families [40]. Due to the availability and accessibility of household resources for single older people living with their children, an adult child (P) is prone to choose family care instead of either institutional care or letting their older parent (G) live alone [41]. Another significant factor is traditional Chinese cultural norms that mandate that older Chinese people live in multigenerational family units [2]. When examining whether to choose formal institutional care, people are likely to prefer informal home care because the evidence suggests that informal and formal care are substitutes [7,42]. Consequently, single older people are unwilling to choose institutional care unless they have no children or have a severe disease [14].

We suggested that single older people, as grandparents, are more likely to co-reside with their grandchildren in parent-maintained homes. Single older people suffer from emotional deprivation; however, grandparents and grandchildren bring a great wealth of emotional and practical support to each other [33]. Based on the demonstration effect and social exchange theory, we then predicted that single older people living with their grandchildren would shape family members’ attitudes. Given these factors and considering that the social exchange effect provides a mechanism for marital status differences in the intergenerational transmission of caregiving norms, we proposed the following hypothesis:

**Hypothesis** **4** **(H4).** *Marital status has potentially diverse effects on institutional care preference*.

### 2.6. The Heterogeneous Effects of Gender

We further suggested that gender differences in intergenerational interactions significantly influence the effect of living with grandchildren on family members’ attitudes toward sending older people to nursing homes. Due to their longer average life expectancies compared to men, women have a higher likelihood of spending their later years without a spouse [27]. Hence, single women have the potential to rely more heavily on their children for support, leading to enhanced motivation to manipulate their children’s preferences under social exchange theory [43]. Older women are more likely to provide childcare when living with their grandchildren, with care exchange expectations for receiving future eldercare from their adult children [29].

**Figure 1:**
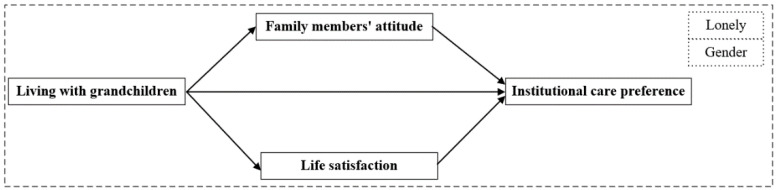
Proposed model of the study.

In addition, socialization enhances gender differences in intergenerational support in the division of labor within a household [43]. According to traditional kin-keeper theory, grandmothers participate more actively in intergenerational interactions and exchanges of support than grandfathers do, thereby creating closer emotional bonds with their family members [21]. In addition, gender differences were discovered in terms of the desired supportive environment, with men preferring group households and women preferring familial living arrangements [14]. In essence, older women living with their grandchildren are more likely to support their grandchildren and other family members. Therefore, according to the social exchange effect, their families are less likely to place them in nursing homes. The proposed model of the study is shown in Figure 1.

**Hypothesis** **5** **(H5).** *Gender has potentially diverse effects on institutional care preference*.

## 3. Materials and Methods

### 3.1. Sample and Data Collection

Our sample drew from the 2014 China Longitudinal Aging Social Survey (CLASS) database, a national, continuous large-scale social survey conducted by the National Survey Research Center at Renmin University of China. The CLASS Baseline Survey was officially launched in 28 provinces (cities and autonomous regions), except Hong Kong, Taiwan, Macau, Hainan, Xinjiang, and Tibet, from August–October 2014. The survey used a stratified multistage probability sampling method. First, the county-level area was chosen as the main sample unit, which includes counties, county-level cities, and districts (PSU). As the secondary sampling unit, the community/village committee was selected (SSU).

CLASS aims to analyze many issues facing the older people as they age and to enhance their quality of life. Study participants were Chinese people aged 60 years or older living in China. The 2014 CLASS examined social and economic factors, including the physical and mental health, intergenerational relationships, and social care situations of the participants. The survey was conducted through face-to-face interviews. A structured questionnaire was read and completed by trained interviewers based on answers provided by the interviewees. The 2014 baseline CLASS survey interviewed 11,511 individuals over 60 years old from 462 communities. This study included a total of 10,339 completed questions on older people’s preferences regarding institutional care. A total of 2,156 older people answered questions about family members’ attitudes toward sending the older people to a nursing home/old age home, so the results of the mediating effect of family members’ attitudes were limited. In total, 48 percent of the older individuals in our sample were male, 52 percent were female, and 33 percent were single. Eighty-four percent of the participants owned their commodity housing.

### 3.2. Measures

#### 3.2.1. Institutional Care Preferences

The dependent variable was older people’s preferences regarding institutional care. Participants were asked to answer the question: “Would you accept living in a nursing home/old age home under circumstances such as poor health, feeling lonely, and others?” This dependent variable was dichotomized (0 = I do not want to live in a nursing home under any conditions, 1 = there are some conditions under which I would like to live in a nursing home) to indicate higher preferences versus lower preferences for institutional care.

#### 3.2.2. Living with Grandchildren

Living with grandchildren was determined by the number of grandchildren currently alive and living with the older person. This was evaluated based on the interviewees’ responses to: “How many grandchildren under the age of 18 years old do you live with?”

#### 3.2.3. Family Members’ Attitudes

The item on family members’ perspectives was self-reported by the participants according to the question: “Would your children be willing to send you to a nursing home/old age home?” The response categories were coded as 1 = would agree to send you to a nursing home/old age home and 0 = would disagree.

#### 3.2.4. Life Satisfaction

The life satisfaction of older people was measured by a global single item. The question item was: “Are you satisfied with your life?” The variable was coded as 1 = satisfied with life and 0 = unsatisfied with life.

#### 3.2.5. Single Older People

The single older individual item was created as a binary variable. The question item was: “What is your current marital status?” The variable was coded such that 1 = single or separated/divorced and 0 = married or partnered.

#### 3.2.6. Gender

The older people were presented with an initial question asking: “What’s your gender?” The results were coded such that 1 = female and 0 = male.

#### 3.2.7. Control Variables

Some researchers have used the Anderson model to integrate the factors affecting older people’s preferences [41]. Accordingly, this paper controlled for the six variables based on the Anderson theoretical model of influencing factors. The forms of the Anderson model consist of three main factors: predisposing characteristics, enabling factors, and demand factors. Age and education level were used to indicate predisposing characteristics, while homeownership, social security benefits, and family economic status, were critical enabling factors, and health status, degree of activities of daily living (ADL), and degree of instrumental activities of daily living (IADL), were regarded as demand factors. All these variables were introduced as control variables [19,38,44]. In line with previous research, the education level of the respondents was presented in categories such that 1 = less than a high school level, 2 = high school level, and 3 = college level and above [45]. ADL and IADL are the two most commonly used indicators to measure physical health status. The study used a nine-item version of the ADL scale and a ten-item version of the IADL scale. These indices were classified into three categories ranging from 1 = the most difficulties and worst physical health to 3 = the least difficulties and best physical health. Homeownership was coded such that 1 = owned commodity housing in destination countries and 0 = owned a house [19]. Social security benefits were regarded as the older Chinese person’s primary income, including enterprise employee basic pressures (EEBP), urban-rural resident social pressures (URRSP), poverty funds, and advanced-age allowances [44,46]. Family economic status was measured by household income over the last year. The researchers calculated the social security benefits and family economic status of the logarithm. The definition of all variables is shown in Table 1.

### 3.3. Analytical Strategy

Binary logit regression was applied to analyze the effects of living with grandchildren on institutional care preferences in China. To test the influence mechanism, this article used the degree of family members’ attitudes and life satisfaction as intermediary variables and constructed mediating effect models to test the influence mechanism. The following reduced form equation served as a benchmark model for assessing institutional care preferences. First, regression model (1) was established. Second, the intermediary variables of family members’ attitudes and life satisfaction were used as the explained variables, and living with grandchildren was used as an explanatory variable to establish regression model (2). Third, the institutional care preference variable was used as the explained variable, and the intermediary variables of family members’ attitudes and life satisfaction were used as explanatory variables to establish regression model (3). Fourth, institutional care preferences, living with grandchildren, and the intermediary variables were included in regression model (4) to observe the whole effect. If coefficients β1, ϕ1, and ρ1, were significant and ω1 either lower than β1 or became less significant, then a mediating effect existed; that is, the theoretical mechanism and research hypothesis described above was established.

Y_i_ = β_0_ + β_1_grandchildren_i_+βX_i_ + ε_i_ (1)

Mid_ij_ = ϕ_0_ + ϕ_1_grandchildren_i_ + ηX_i_ + λ_i_ (2)

Y_i_ = ρ_0 +_ ρ_1_Mid_ij_+γX_i_ + ψ_i_ (3)

Y_i_ = ω_0_ + ω_1_grandchildren_i_ + ω_2_Mid_i1_ + ω_2_Mid_i2_ + φX_i_ + τ_i_ (4)

## 4. Results

### 4.1. Baseline Effects

We used the stepwise regression method to test our hypotheses, as shown in Table 2. We evaluated the marginal effects of living with grandchildren, family members’ attitudes, life satisfaction, and control variables, on the probabilities of institutional care preferences. In Table 2, column (1) shows the main effects of institutional care preferences. Based on this result, H1 was supported (β = −0.0278, S.E. = 0.0079, ρ < 0.001), suggesting that living with one more grandchild significantly decreased the institutional care preference by 2.78%. Column (2) shows that living with grandchildren was significantly negatively related to family members’ attitudes (β = −0.0358, S.E. = 0.0189, ρ < 0.05), meaning that living with one additional grandchild reduced family members’ attitudes toward sending older people to nursing homes by 3.58%. Column (3) shows the significantly positive effect of family members’ attitudes on institutional care preferences (β = 0.1838, S.E. = 0.0378, ρ < 0.001), as predicted in H2. Moreover, column (4) shows that living with one additional grandchild would foster older people’s life satisfaction by 1.12%, and H3 was supported (β = 0.0112, S.E. = 0.0067, ρ < 0.05). Column (5) shows the proposition of a negative relationship between older people’s life satisfaction and institutional care preferences (β = −0.0640, S.E. = 0.0117, ρ < 0.001). Finally, consistent with H2 and H3, we put all independent variables, control variables, and mediating variables together into model (6) to consider their simultaneous effects, representing a more robust and conservative approach, predicting that family members’ attitudes and life satisfaction fully mediated the relationship between living with grandchildren and institutional care preferences.

### 4.2. Heterogeneous Effects

To assess the difference effect, we divided our sample into several subgroups to explore the heterogeneous effects of institutional care preferences. We split the sample by marital status into single older people and older people with a spouse.

The estimation shown in Table 3 column (2) reveals that a negative relationship was supported between living with grandchildren and institutional care preferences for older people with spouses (β = −0.0270, S.E. = 0.0097, ρ < 0.01), while there was a larger significant effect in the single older population, which is shown in column (1) (β = −0. 0317, S.E. = 0.0137, ρ < 0.05). Columns (3) and (4) verify a negative association between living with grandchildren and family members’ attitudes among single older people and older people with spouses. Only the negative effect for single older people was significant; that is, a single older person living with one additional grandchild had a lower possibility of 13.37% of family members agreeing to send them to an institutional care facility (β = −0. 1337, S.E. = 0.0467, ρ < 0.01). According to columns (5) and (6), the results suggested that family members’ attitudes had a greater impact on institutional care preferences for single older people (β = 0.4384, S.E. = 0.1274, ρ < 0.001) than for older people with spouses (β = 0.1570, S.E. = 0.0445, ρ < 0.001). The results in columns (8) and (10) show that life satisfaction is significantly mediated between living with grandchildren and institutional care preferences in the group of older people with spouses. Living with grandchildren boosted their life satisfaction by 1.14% (β = 0.0114, S.E. = 0.0800, ρ < 0.05), while institutional care preferences decreased by 5.49% (β = −0.0549, S.E. = 0.0149, ρ < 0.001). This suggests that single older people who live with their grandchildren are more dependent on family advice, while older people with spouses who live with their grandchildren have higher life satisfaction and are less likely to choose a nursing home for care. These results were consistent with H4.

Table 4 presents estimations from different gender groups, which shows a robust consistent conclusion with the benchmark estimation. Columns (1) and (2) show institutional care preferences as the dependent variable, with females and males as the research sample, respectively. These results show that living with grandchildren induced institutional care preferences in both female (β = −0.0298, S.E. = 0.0110, ρ < 0.01) and male (β = −0.0304, S.E. = 0.0114, ρ < 0.01) groups. Columns (3) and (4) show family members’ attitudes as the dependent variable. The coefficient of the female group was significant (β = −0.1055, S.E. = 0.0299, ρ < 0.001), while that of the male group was not significant (β = 0.0379, S.E. = 0.0290, ρ > 0.1), meaning that the effect of living with grandchildren on promoting family members’ attitudes was more pronounced in female than in male older adults The results in columns (5) and (6) show that in female older adults, family members agreeing to send the older person to a nursing home boosted institutional preferences by 34.67% (β = 0.3467, S.E. = 0.1052, ρ < 0.001), while in male older adults, it increased by 17.40% (β = 0.1740, S.E. = 0.0548, ρ < 0.01). These findings confirm that for female older adults, the impact of family members agreeing to send them to institutional care facilities compared to living with grandchildren played a considerable role, while male older adults were not affected. Columns (7)–(10) show the mediating role of life satisfaction in the model. The coefficients of the independent variable (living with grandchildren) among the female older group shown in column (7) were significantly positive (β = 0.0072, S.E. = 0.0095, ρ < 0.05), while the coefficients of the male older group shown in column (8) were not significant (β = 0.0113, S.E. = 0.0096, ρ > 0.1). Columns (9) and (10) show institutional preference as the dependent variable, and the coefficients of both the female (β = −0.0634, S.E. = 0.0164, ρ < 0.001) and male older groups (−β = 0.0755, S.E. = 0.0169, ρ < 0.001) were significant, meaning that life satisfaction played a mediating role in the relationship between living with grandchildren and institutional care preferences in the older female group. These results imply that older female adults gain more value from family members and life satisfaction, and hypothesis H5 was supported.

## 5. Theoretical Implications

This study makes several valuable contributions to the literature. First, our findings reflected intergenerational reciprocity, which helps to explain the intergenerational exchange involved in family eldercare arrangements and extends intergenerational exchanges from two to three generations, signifying the cyclical nature of interchange [15]. In addition to intergenerational support in terms of exchanges of resources and resulting obligations, we cannot overlook emotional attachment in ‘collectivist’ societies [16,22]. The findings may be more reflective of ‘collectivist’ societies (e.g., Asian, African, South American, Pacific Islander, parts of Scandinavian), rather than ‘individualistic’ societies (e.g., European, parts of Scandinavian, North American, Australiana). This study showed that situations in which older people lived with their grandchildren were positively related to their life satisfaction. This represents downwards (G to C) transfers where rewards are sought in the form of higher life quality [31]. This result shows a form of investment through intergenerational exchanges of support motivated by obligations. The evidence also confirmed that adult children’s attitudes were positively related to older people’s preferences for their care, which is an example of the transmission of an intergenerational trusting relationship. Furthermore, this paper found that the demonstration and intergenerational exchanges in the transmission of intergenerational reciprocity are not limited to two adjacent generations; the transmission can take place in three or more generations. In this way, the cyclical nature of reciprocity contributes to the development of the understanding of intergenerational exchange support.

Second, our work promotes the demonstration effect by addressing how and why older people co-reside with their grandchildren, and their family members’ subsequent attitudes toward eldercare arrangements. It is important to note that the motives for human behavior are sophisticated and cannot be listed individually [43]. In addition to the social exchange motives often discussed in the literature, the demonstration effect may be another motivation behind intergenerational relationships. In this regard, the middle generation, adult parents (P), regarded co-residing with and caring for older grandparents (G) at home as providing an example, anticipating that other family members would perceive taking responsibility for eldercare as the right thing to do [27]. In this way, according to the demonstration effect, adult parents’ demonstrations of caregiving can be duplicated later by their children, meaning that these adult parents (P) would be cared for in their later life. That is why adult parents (P) with minor children (C) prefer informal care at home rather than institutional care. Some scholars have focused on intergenerational exchanges within the family, but studies have rarely indicated the demonstration effect in this relationship. This study extends the current state of eldercare literature and makes a substantial advancement on the demonstration effect [27,28,32,43].

Third, our findings support the importance of gender differences by showing the apparent effects of co-residing with grandchildren on family members’ attitudes toward eldercare preferences. In line with previous evidence, our results showed that traditional gender roles are salient [47], especially regarding more age characteristics, family function, and family intimacy. Traditionally, women have played the role of primary family caregivers, especially in Confucian cultures in Eastern countries [18]. Compared to men, women take on more responsibility for household chores and provide more care for grandchildren. The broader implications of these findings are that older female adults tend to live with adult children and care for their grandchildren, creating a more profound sense of gratitude in adult children [43]. In summary, family members of single older female adults prefer to informal care, and that they do not want to place them in institutional care if they are living with their grandchildren because they can provide care.

Finally, we extended and emphasized cultural models and living arrangements regarding how senior care obligation plays a crucial role in reinforcing the effects of intergenerational exchange and demonstration in the Chinese context. Intergenerational co-residence has been defined as part of the virtue of filial piety, which persists in China [21,43]. More specifically, co-residence based on both social exchange and the demonstration effect can be seen as mutual assistance, which is, in essence, a type of informal care. It appears that the behavior of Chinese individuals tends to follow the intergenerational exchange and the demonstration effect because importance is attached to social norms [22,29]. Generally, this article has contributed to the literature on family relationships in China.

## 6. Practical Implications

Our research also offers various implications for eldercare in practice. First, our study broadly indicated that older people prefer informal family care over institutional care because of the scarcity and imperfection of the national LTC system of eldercare, especially that of formal institutional care. This is the same result as a previous study in which more than 50 percent of respondents from the general population preferred informal care at home [48]. At this time, family size has shrunk due to the one-child policy formulated in the 1970s and the migration patterns of young people leaving their older parents, resulting in single seniors finding it more difficult to obtain home care [17]. As such, for the sake of improving the acceptance of and attitudes surrounding institutional social services for older adults and their family members, we recommend that the government and social media energetically publicize the advantage of integrated social and institutional care services and conduct training for professional caregivers [2]. The government can also encourage nursing homes to develop appropriate financing, enrich comprehensive care service content, and improve their quality of service [15,41], which will benefit older adults’ well-being and help break the social prejudices of institutional care.

Our empirical study also pointed out the importance of intergenerational redistribution and reciprocal exchange. Our findings showed that single older people were more likely to live with their family members than in institutional care, perhaps indicating that children and family members are better care providers in terms of several ailments in older people, such as dementia or Alzheimer’s disease [27]. Importantly, children are always the primary informal caregivers of their single older parents, replacing spouses according to the Chinese cultural norm of filial piety [17]. Conversely, younger single older people can help their children take care of their young grandchildren [46]. Therefore, it is essential to emphasize that responsibility and informal care should be shared in integrated family networks. Moreover, in addition to formal institutional care services, in-home aging policies should be launched to support households with single older people by offering respite training in communities and eldercare services at home.

## 7. Limitations and Future Research

As with most empirical research, our study had four limitations that provide potential directions for future research. First, our study relied on cross-sectional data, making it difficult to infer causality underlying our hypotheses or intergenerational exchange among three generations through different phases of the family life cycle [21,40]. Through observations over time, the changing characteristics of older people could support families through demonstration and intergenerational exchange effects. This could be done using a longitudinal field study to understand how different factors predict changes in single older people’s demands for appropriate living arrangements and their causal impact on institutional care preferences.

Second, the data we collected are only from the perspectives of older people. Although our findings that single older people prefer to live with their family members rather than to be cared for in nursing homes align with previous work [21], we did not capture the relationship between single older people and care preferences from the perspective of the middle generation, which is one of the key decision-makers. Moreover, fewer interviewees answered the mediating variable of family members’ attitudes than answered the other variables. In particular, the older generation was more likely to view themselves as closer to the younger generation due to the generational-stake phenomenon [33]. Thus, the investigation of family networks from the perspectives of different generations may provide a clearer picture of how three or more generations create a family exchange of support. To address this concern, a future investigation could involve respondents who are older people, their adult children, and their grandchildren to provide more nuanced findings. Furthermore, future research needs to consider whether one adult child, more than one adult child, or the spouse of an adult child participates in care services [32], and how their actions affect grandchildren.

Finally, this study relied on a theoretical framework, with family relationship-based constructions of the demonstration effect and reciprocal exchange theories, although the extent of intergenerational reciprocity varies across cultural settings [19]. Even though both intergenerational exchange and demonstration effects benefiting eldercare arrangements for one’s family can be considered universal, socially focused values [49], Asian cultural norms may emphasize these values more than other cultures do. Therefore, it is essential to note that cultural factors may affect the generalizability of our findings. Our research and previous literature on family exchanges and demonstration motivation were conducted in Eastern Asian countries; future studies could explore whether our results could be replicated in Latin American or European countries [40]. A cross-national study could target eldercare preferences in different cultural contexts, especially demonstration and intergenerational exchange relationships between adult parents’ attitudes, grandchildren’s demands, and older parents’ care preferences.

## 8. Conclusions

To conclude, our results prove the salience of linking living with grandchildren, family members’ attitudes, and eldercare preferences with caring motives, namely, intergenerational exchange and demonstration effects. These prevailing motivations offered evidence about the interconnection of several generations’ motivations for support and care behaviors, suggesting that family exchange relationships significantly affect living arrangements and family members’ attitudes toward eldercare, thus transmitting eldercare preferences. We therefore extended the theoretical knowledge of older people’s care preferences under social exchange and demonstration theories, and the findings may be more reflective of emotional attachment in ‘collectivist’ societies, compared to ‘individualistic’ societies, which had an additional impact on public service implications.

## Figures and Tables

**Table 1:** Definition of variables.

Variables	Operationalization	Mean	SD
Institutional care preference	1 = there are some conditions under which I would like to live in a nursing home, 0 = I do not want to live in a nursing home under any conditions	0.28	0.45
Living with grandchildren	Number of grandchildren under the age of 18 years old you live with	0.30	0.67
Family members’ attitudes	1 = agree to send you to a nursing home/old age home, 0 = disagree	0.29	0.45
Life satisfaction	1 = satisfied with life, 0 = unsatisfied with life	0.75	0.43
The single older	1 = single or separated/divorced and 0 = married or partnered	0.33	0.47
Gender	1 = female, 0 = male	0.52	0.50
Age	Measured in years	70.7	8.14
Education	1 = less than a high school level, 2 = high school, and 3 = college and above	1.22	0.54
ADL	Degree of activity of daily living	1.07	0.24
IADL	Degree of instrumental activity of daily living	1.20	0.37
Homeownership	1 = owned commodity housing in destination counties, 0 = others	0.84	0.37
Social security benefits	older people’s primary income, including Enterprise Employee Basic Pensions (EEBP), Urban-Rural Resident Social Pensions (URRSP), poverty funds, and advanced-age allowances	5.99	1.72
Family economic status	Household income over the last year	9.10	1.41

SD = standard deviation.

**Table 2:** Marginal effect on determinants of institutional care preference.

Variables	Institutional Care Preference	Family Members’ Attitude	Institutional Care Preference	Life Satisfaction	Institutional Care Preference	Institutional Care Preference
	(1)	(2)	(3)	(4)	(5)	(6)
Living with grandchildren	−0.0278 ***	−0.0358 *		0.0112 *		0.0171
	(0.0079)	(0.0189)		(0.0067)		(0.0218)
Family members’ attitude			0.1838 ***			0.1685 ***
			(0.0378)			(0.0369)
Life satisfaction					−0.0640 ***	−0.0622 *
					(0.0117)	(0.0307)
Age	−0.0074 ***	−0.0039 *	−0.0053 ***	0.0068 ***	−0.0068 ***	−0.0048 ***
	(0.0007)	(0.0015)	(0.0014)	(0.0006)	(0.0007)	(0.0014)
Education	0.0211*	0.0142	0.0109	0.0089	0.0237 **	0.0153
	(0.0088)	(0.0176)	(0.0178)	(0.0091)	(0.0088)	(0.0179)
ADL	0.0420	0.0291	−0.0891	−0.0648 *	0.0470	−0.0912
	(0.0377)	(0.0657)	(0.0638)	(0.0296)	(0.0387)	(0.0628)
IADL	−0.0346	−0.0098	0.0109	−0.1479 ***	−0.0474 +	−0.0030
	(0.0252)	(0.0543)	(0.0522)	(0.0198)	(0.0256)	(0.0512)
Homeownership	0.0238	0.0194	−0.0313	−0.0027	0.0254 +	−0.0439
	(0.0150)	(0.0362)	(0.0344)	(0.0127)	(0.0150)	(0.0353)
Social security benefits	0.0495 ***	0.0478 **	−0.0031	0.0145 **	0.0519 ***	0.0038
	(0.0056)	(0.0154)	(0.0135)	(0.0049)	(0.0056)	(0.0133)
Family economic status	−0.0123 +	0.0040	−0.0216	0.0240 ***	−0.0112 +	−0.0261 +
	(0.0065)	(0.0176)	(0.0159)	(0.0054)	(0.0065)	(0.0158)
Provincial FE	Yes	Yes	Yes	Yes	Yes	Yes
N	8232	1752	950	8876	8177	940
Pseudo R2	0.1230	0.1036	0.2275	0.0916	0.1245	0.2443

Note: Standard errors in parentheses. + *p* < 0.1, * *p* < 0.05, ** *p* < 0.01, *** *p* < 0.001.

**Table 3:** Marginal effect on heterogeneous effects of single older people: determinants of institutional care preference.

Variables	Institutional Care Preference		Family Members’ Attitude		Institutional Care Preference		Life Satisfaction		Institutional Care Preference		Institutional Care Preference	
	Single	With Spouse	Single	With Spouse	Single	With Spouse	Single	With Spouse	Single	With Spouse	Single	With Spouse
	(1)	(2)	(3)	(4)	(5)	(6)	(7)	(8)	(9)	(10)	(11)	(12)
Living with grandchildren	−0.0317 *	−0.0270 **	−0.1337 **	−0.0156			0.0075	0.0114 *			−0.0693	0.0538
	(0.0137)	(0.0097)	(0.0467)	(0.0230)			(0.0129)	(0.0800)			(0.0600)	(0.0436)
Family members’ attitude					0.4384 ***	0.157 0 ***					0.4517 ***	0.1424 ***
					(0.1274)	(0.0445)					(0.1240)	(0.0424)
Life satisfaction									−0.0750 ***	−0.0549 ***	−0.1313	−0.0784
									(0.0188)	(0.0149)	(0.0800)	(0.0488)
Age	−0.0066 ***	−0.0074 ***	−0.0064 *	−0.0028	−0.0060	−0.0050 **	0.0063 ***	0.0078 ***	−0.0057 ***	−0.0068 ***	−0.0063	−0.0042 *
	(0.0011)	(0.0009)	(0.0031)	(0.0021)	(0.0049)	(0.0019)	(0.0011)	(0.0009)	(0.0011)	(0.0009)	(0.0052)	(0.0019)
Education	0.0363 *	0.0156	0.0820 +	0.0135	0.1413	−0.0011	0.0023	0.0042	0.0366*	0.0180 +	0.1547	0.0016
	(0.0180)	(0.0104)	(0.0466)	(0.0211)	(0.1065)	(0.0210)	(0.0212)	(0.0102)	(0.0180)	(0.0104)	(0.0947)	(0.0211)
ADL	0.0415	0.0300	0.0097	−0.0143	−0.3033 +	0.0201	−0.0652	−0.0530	0.0464	0.0343	−0.2201	0.0095
	(0.0515)	(0.0532)	(0.1067)	(0.1008)	(0.1738)	(0.0891)	(0.0443)	(0.0414)	(0.0528)	(0.0546)	(0.1756)	(0.0909)
IADL	−0.0542	−0.0055	0.0436	0.0136	0.1558	−0.0809	−0.1264 ***	−0.1801 ***	−0.0694 *	−0.0169	0.0185	−0.0912
	(0.0347)	(0.0358)	(0.0934)	(0.0779)	(0.1524)	(0.0729)	(0.0300)	(0.0277)	(0.0353)	(0.0363)	(0.1582)	(0.0733)
Homeownership	0.0241	0.0123	−0.0252	0.0381	−0.0622	−0.0677	−0.0279	0.0236	0.0249	0.0146	−0.0987	−0.0864
	(0.0204)	(0.0214)	(0.0638)	(0.0523)	(0.0861)	(0.0536)	(0.0199)	(0.0171)	(0.0203)	(0.0215)	(0.0863)	(0.0545)
Social security benefits	0.0355 ***	0.0566 ***	0.0480	0.0567 **	−0.0096	−0.0127	0.0063	0.0161 **	0.0389 ***	0.0588 ***	0.0241	−0.0040
	(0.0101)	(0.0068)	(0.0359)	(0.0191)	(0.0420)	(0.0188)	(0.0098)	(0.0056)	(0.0101)	(0.0069)	(0.0403)	(0.0194)
Family economic status	−0.0023	−0.0144 +	0.0412	−0.0144	−0.0294	−0.0174	0.0274 **	0.0239 ***	−0.0026	−0.0134	−0.1053 +	−0.0194
	(0.0108)	(0.0082)	(0.0389)	(0.0228)	(0.0525)	(0.0214)	(0.0102)	(0.0064)	(0.0109)	(0.0082)	(0.0626)	(0.0221)
Provincial FE	Yes	Yes	Yes	Yes	Yes	Yes	Yes	Yes	Yes	Yes	Yes	Yes
N	2694	5505	409	1197	409	1197	2822	5992	2672	5472	409	1197
Pseudo R2	0.1235	0.1254	0.1549	0.0883	0.3512	0.2160	0.1181	0.0931	0.1261	0.1263	0.4036	0.2384

Note: Standard errors in parentheses. + *p* < 0.1, * *p* < 0.05, ** *p* < 0.01, *** *p* < 0.001.

**Table 4:** Marginal effect on heterogeneous effects of gender: determinants of institutional care preference.

Variables	Institutional Care Preference		Family Members’ Attitude		Institutional Care Preference		Life Satisfaction		Institutional Care Preference		Institutional Care Preference	
	Female	Male	Female	Male	Female	Male	Female	Male	Female	Male	Female	Male
	(1)	(2)	(3)	(4)	(5)	(6)	(7)	(8)	(9)	(10)	(11)	(12)
Living with grandchildren	−0.0298 **	−0.0304 **	−0.1055 ***	0.0379			0.0072 *	0.0113			0.0140	0.0215
	(0.0110)	(0.0114)	(0.0299)	(0.0290)			(0.0095)	(0.0096)			(0.0362)	(0.0424)
Family members’ attitude					0.3467 ***	0.1740 **					0.3088 **	0.1610 **
					(0.1052)	(0.0548)					(0.0955)	(0.0542)
Life satisfaction									−0.0634 ***	−0.0755 ***	−0.1078 +	−0.0737
									(0.0164)	(0.0169)	(0.0569)	(0.0591)
Age	−0.0082 ***	−0.0072 ***	−0.0053 *	−0.0028	−0.0102 ***	−0.0067 **	0.0061 ***	0.0075 ***	−0.0075 ***	−0.0066 ***	−0.0089 **	−0.0059 *
	(0.0010)	(0.0010)	(0.0023)	(0.0024)	(0.0029)	(0.0025)	(0.0009)	(0.0009)	(0.0010)	(0.0010)	(0.0029)	(0.0026)
Education	0.0345 *	0.0189	0.0662 *	−0.0160	0.0401	−0.0165	0.0327 *	−0.0003	0.0379 **	0.0221 +	0.0666	−0.0151
	(0.0137)	(0.0119)	(0.0279)	(0.0256)	(0.0380)	(0.0314)	(0.0156)	(0.0113)	(0.0137)	(0.0118)	(0.0421)	(0.0310)
ADL	0.0052	0.0718	0.0044	0.0283	−0.2627 +	−0.4906 +	−0.0564	−0.0834 +	0.0071	0.0765	−0.2302	−0.4516 +
	(0.0498)	(0.0593)	(0.0899)	(0.1239)	(0.1436)	(0.2507)	(0.0372)	(0.0493)	(0.0512)	(0.0606)	(0.1469)	(0.2486)
IADL	−0.0118	−0.0336	0.0701	−0.0773	0.0820	0.2142	−0.1418 ***	−0.1593 ***	−0.0227	−0.0514	0.0331	0.1593
	(0.0337)	(0.0388)	(0.0793)	(0.0926)	(0.1124)	(0.1534)	(0.0265)	(0.0309)	(0.0344)	(0.0392)	(0.1151)	(0.1498)
Homeownership	0.0508 *	−0.0151	0.0260	0.0172	−0.0118	0.0021	−0.0010	0.0002	0.0569 **	−0.0177	−0.0071	−0.0381
	(0.0200)	(0.0229)	(0.0532)	(0.0566)	(0.0717)	(0.0631)	(0.0167)	(0.0198)	(0.0201)	(0.0230)	(0.0670)	(0.0699)
Social security benefits	0.0475 ***	0.0504 ***	0.0287	0.0613 **	−0.0117	0.0218	0.0157 *	0.0119 +	0.0503 ***	0.0525 ***	0.0002	0.0263
	(0.0084)	(0.0077)	(0.0268)	(0.0214)	(0.0261)	(0.0246)	(0.0074)	(0.0065)	(0.0084)	(0.0077)	(0.0266)	(0.0238)
Family economic status	−0.0074	−0.0140	0.0391	−0.0136	−0.0402	−0.0307	0.0203 **	0.0342 ***	−0.0060	−0.0126	−0.0438	−0.0414
	(0.0093)	(0.0094)	(0.0288)	(0.0264)	(0.0316)	(0.0265)	(0.0077)	(0.0077)	(0.0093)	(0.0095)	(0.0320)	(0.0270)
Provincial FE	Yes	Yes	Yes	Yes	Yes	Yes	Yes	Yes	Yes	Yes	Yes	Yes
N	4122	4053	807	806	807	806	4500	4373	4094	4026	807	806
Pseudo R2	0.1532	0.1099	0.1400	0.1042	0.3239	0.2475	0.1027	0.1084	0.1544	0.1126	0.3462	0.2743

Note: Standard errors in parentheses. + *p* < 0.1, * *p* < 0.05, ** *p* < 0.01, *** *p* < 0.001.

## Data Availability

Data use of the 2014 China Longitudinal Aging Social Survey (CLASS) is supported and approved by the National Survey Research Center at Renmin University of China of China. Official website: http://class.ruc.edu.cn/ (accessed on 5 May 2020).
